# Comparison of gene expression microarray data with count-based RNA measurements informs microarray interpretation

**DOI:** 10.1186/1471-2164-15-649

**Published:** 2014-08-04

**Authors:** Arianne C Richard, Paul A Lyons, James E Peters, Daniele Biasci, Shaun M Flint, James C Lee, Eoin F McKinney, Richard M Siegel, Kenneth GC Smith

**Affiliations:** Cambridge Institute for Medical Research and Department of Medicine, University of Cambridge, Cambridge, UK; Immunoregulation Section, Autoimmunity Branch, National Institute of Arthritis and Musculoskeletal and Skin Diseases, Bethesda, MD USA

**Keywords:** Microarray, NanoString, nCounter, Gene expression

## Abstract

**Background:**

Although numerous investigations have compared gene expression microarray platforms, preprocessing methods and batch correction algorithms using constructed spike-in or dilution datasets, there remains a paucity of studies examining the properties of microarray data using diverse biological samples. Most microarray experiments seek to identify subtle differences between samples with variable background noise, a scenario poorly represented by constructed datasets. Thus, microarray users lack important information regarding the complexities introduced in real-world experimental settings. The recent development of a multiplexed, digital technology for nucleic acid measurement enables counting of individual RNA molecules without amplification and, for the first time, permits such a study.

**Results:**

Using a set of human leukocyte subset RNA samples, we compared previously acquired microarray expression values with RNA molecule counts determined by the nCounter Analysis System (NanoString Technologies) in selected genes. We found that gene measurements across samples correlated well between the two platforms, particularly for high-variance genes, while genes deemed unexpressed by the nCounter generally had both low expression and low variance on the microarray. Confirming previous findings from spike-in and dilution datasets, this “gold-standard” comparison demonstrated signal compression that varied dramatically by expression level and, to a lesser extent, by dataset. Most importantly, examination of three different cell types revealed that noise levels differed across tissues.

**Conclusions:**

Microarray measurements generally correlate with relative RNA molecule counts within optimal ranges but suffer from expression-dependent accuracy bias and precision that varies across datasets. We urge microarray users to consider expression-level effects in signal interpretation and to evaluate noise properties in each dataset independently.

**Electronic supplementary material:**

The online version of this article (doi:10.1186/1471-2164-15-649) contains supplementary material, which is available to authorized users.

## Background

Over the last decade, gene expression microarrays have become a common tool for examining gene transcript levels in hypothesis-free investigations. Microarray data is used for a wide variety of analyses, such as unsupervised clustering, classification, differential expression analysis, and expression quantitative trait loci mapping (as reviewed in [1]). These studies aim to differentiate subtle changes in relevant features from other biological and technical variation. Sample preparation for gene expression microarray requires one or more transcriptional steps, followed by labelling, hybridization, and intensity measurement [2]. At each step, technical variations and accompanying biases are introduced.

Microarray data preprocessing and batch correction are important strategies for minimizing such confounding. Preprocessing consists of three steps: background correction, normalization, and summarization; and the choice of methods for these steps can dramatically change experiment results [3]. Preprocessing algorithms must contend with differing probe hybridization efficiencies that result in greater inter-probe than inter-sample variability, probe intensity variances that change with intensity levels, and inter-sample technical error. In comparative studies with spike-in and dilution datasets [4–7], quantile normalization has performed consistently well, and thus robust multi-array average (RMA), using a global background correction, quantile normalization, and a linear model fit by median polish for probe summarization [8], has become a popular method for single-color microarray preprocessing. Even after normalization, variation in processing technician, location and time can result in probe-specific batch effects (as reviewed in [9]). Many algorithms attempt to normalize between batches, some performing location-scale adjustments based on known batch delineations, and others using global matrix factorization under the assumption that technical effects will outweigh biological effects (as comprehensively surveyed in [10]). Comparisons of batch effect removal methods [11, 12] have found the location-scale adjustment empirical Bayesian method ComBat [13] to be robust, particularly when the study does not include multiple platforms or tissue-types, and when cross-batch reference samples are unavailable [12]. ComBat borrows information across genes with similar within-batch expression profiles to correct batch effects while preserving biological covariates, allowing good performance even with small sample sizes [13].

These data processing methods enable comparisons of individual genes between samples, but they leave direct interpretation of the normalized values somewhat ambiguous. This is particularly problematic for genes with low log-transformed expression values, where it can be difficult to differentiate true expression from background noise. After quantile normalization there is no direct calculation of the real expression difference indicated by a log-fold-change in microarray expression values. Common validation technologies, such as quantitative polymerase chain reaction (qPCR, reviewed in [14]) and multiplex branched DNA assay [15], also introduce noise through transcript or signal amplification and analog detection: qPCR measures real-time changes in the level of targeted transcripts during amplification through fluorescence changes and extrapolates their relative concentrations, while branched DNA assays employ a forked hybridization detection scheme, amplifying reporter fluorescence of hybridized probes for signal detection. In addition, although many biological studies use qPCR for validation of significant findings (as sampled by [16]), they examine a limited number of genes selected for specific expression characteristics and thereby fail to provide a global representation from which microarray data properties might be discerned.

Previous studies with spike-in and dilution datasets [5, 6, 17, 18] have demonstrated compression of microarray values at high and low levels of expression. However, a number of studies (summarized by [19]) have raised concerns that spike-in and dilution datasets create highly-controlled background noise and are therefore unlikely to reflect the differing levels of cross-hybridizing molecules within real biological samples [20]. In addition, these constructed “truth” samples often contain large target gene variances that may not represent the subtle changes examined in certain experimental settings. Several studies have used samples from biological tissue to compare multiple microarray platforms with other measurement technologies, either mixing RNA from two tissues at known ratios [21] or harvesting RNA in stimulated and unstimulated conditions [22, 23]. However, these datasets have dramatic target gene variances, the studies compare microarray measurements with those from PCR- and branched-DNA-based technologies that may introduce bias in amplification or detection steps, and their analyses mainly address differential expression discovery rather than global properties of the microarray. To our knowledge, no study has made a systematic examination of microarray signal detection accuracy and precision with diverse biological samples in reference to an amplification-free, digital RNA measurement.

Here we have used a count-based transcript detection technology to address some of the questions raised above, comparing measurements from the Affymetrix Human Gene 1.1 ST microarray with those from the NanoString Technologies nCounter Analysis System [24]. In contrast to qPCR and branched DNA assays, the nCounter Analysis System directly enumerates specific RNA molecules by dual probe hybridization, requiring amplification of neither RNA nor signal. To avoid hybridization and fluorescence scale biases, nCounter reactions are carried out with a great excess of transcript-specific probes before purification, and measurements are made on a digital instead of analog scale. Additionally, because it does not require transcription, the nCounter system protocol never heats samples sufficiently to denature genomic DNA, avoiding contamination from genomic DNA hybridization to oligonucleotide probes (a noted source of noise for qPCR [25]). Finally, nCounter data has recently been shown to be highly robust to different normalization methods [26], providing reassurance that our gold-standard does not suffer computational processing biases. Thus, we interpret ratios of test and control gene counts from the nCounter as true measures of the relative expression of these genes in our samples. In this study, we compared nCounter with microarray expression measurements of an experimental dataset composed of leukocyte subset RNA from healthy controls and patients with either anti-neutrophil-cytoplasmic-antibody-associated vasculitis (AAV) or inflammatory bowel disease (IBD) to exemplify the level of biological variation likely to be encountered in microarray experiments. Our analyses provide a better understanding of how preprocessed microarray results reflect RNA levels in diverse biological samples, translating microarray expression value differences into molecular changes between samples, and highlighting tissue-specific noise properties.

## Methods

### RNA samples

This study was approved by the Cambridgeshire 3 Research Ethics Committee (08/H0306/21) and all individuals provided written informed consent. Samples were from healthy controls and individuals with active, untreated disease with the following diagnoses: IBD, specifically Crohn’s disease (CD) and ulcerative colitis (UC); and AAV, specifically granulomatosis with polyangiitis (GPA) and microscopic polyangiitis (MPA). Whole blood was collected and separated into peripheral blood leukocyte subsets as previously described [27–29]. Briefly, whole blood was passed over a Histopaque 1077 (Sigma-Aldrich) gradient. Red blood cells from the granulocyte-red-blood-cell pellet were lysed in a buffer of 155 mM ammonium chloride, 12 mM sodium bicarbonate, and 100 mM EDTA, and CD16+ neutrophils were separated by magnetic bead-based positive selection (Miltenyi Biotec). Peripheral blood mononuclear cells were split into two fractions for positive selection (Miltenyi Biotec) of CD14+ monocytes and CD19+ B cells. Negative fractions from the CD14+ and CD19+ selections were then used in a second round of positive selection for CD4+ T cells and CD8+ T cells (Miltenyi Biotec), respectively. RNA was extracted with the RNEasy Mini or AllPrep DNA/RNA Mini kit (Qiagen), following the manufacturer’s protocols. This study uses the CD16+ neutrophil, CD14+ monocyte and CD4+ T cell subsets from this dataset, referred to as the CD16, CD14 and CD4 datasets, respectively.

### Microarray

Aliquots of 200 ng total RNA were amplified and labelled for Human Gene 1.1 ST 96-Array (Affymetrix) using the Ambion WT Expression Kit and GeneChip WT Terminal Labeling and Controls Kit (Affymetrix), according to the manufacturer’s protocols. Samples were run on a GeneTitan Multi-Channel (MC) Instrument (Affymetrix) as part of a larger dataset acquired over multiple years. For comparison with nCounter data, we selected only batches with at least 10 samples from the desired cohorts covering at least two different diagnoses to ensure batch correction was resistant to outliers and confounding structure. Microarray data for samples used in the nCounter comparison have been deposited in ArrayExpress with Accession Number E-MTAB-2452.

### Microarray data processing

Gene expression microarrays were filtered for sex discordance and global dimness before data processing. Because the robustness of microarray normalization improves with the number of samples included, arrays were normalized in large, cell-type-specific batches, including all available samples from the selected batches with diagnoses tested in this study (See Additional file 1 for a tabulated summary of arrays included in this processing). Samples were preprocessed with RMA using the oligo Bioconductor package [30] with pd.hugene.1.1.st.v1 [31] annotation. Batch correction was performed using the ComBat function of the sva Bioconductor package [32] specifying diagnosis, sex, and age as covariates to avoid removal of biological differences. Quality control was performed with the arrayQualityMetrics Bioconductor package [33].

### nCounter control gene choice

Control genes for the nCounter Analysis System were chosen for each cell type on the basis of consistent expression across samples in the large, cell-type-specific microarray datasets described above. RMA-preprocessed datasets were separated by batch, and the variance of each gene calculated across all samples. Gene variances were then averaged across batches. Examining average variance versus mean expression revealed low variance at very high expression levels, suggesting array saturation or preprocessing compression effects (see Additional file 2). In order to select control genes that were well-expressed but not completely saturated, we filtered for genes with mean microarray expression values between 8 and 12 (see Additional file 2 for variance-expression relationship). These genes were then sorted by average within-batch variance, and control genes were chosen from the 2% with the lowest variance based on functional annotation suggesting stable, high expression in leukocytes (see Additional file 3). The use of two control genes per dataset was modelled after Reis et al. [34].

### nCounter

RNA was prepared for and run on the nCounter Analysis System (NanoString Technologies), according to the manufacturer’s protocol in a total of 6 runs over 8 days. To test for RNA degradation, several sentinel samples from each cell-type-specific dataset were examined by 2100 Bioanalyzer (Agilent Technologies, Inc) according to the manufacturer’s protocol. All sentinel samples were confirmed to be of good quality with RIN ≥ 9.5. One sample was duplicated in a separate nCounter run to evaluate reproducibility and run-specific effects (see Additional file 4). RNA was loaded at 100 ng per sample with the exception of one sample with low RNA yield; this was run at 59 ng and did not result in a low-count quality control flag. All hybridizations were 17 hours long, and all counts were gathered by scanning on HIGH mode for 280 fields of view per sample.

### nCounter data processing

No nCounter samples were flagged by nSolverAnalysisSoftware (NanoString Technologies) for quality control. nCounter data was normalized for hybridization and counting efficiency in cell-type specific groups. Each sample was multiplicatively normalized to have the same geometric mean of nCounter-provided positive control probe counts. No normalization factors were outside the NanoString-recommended range of 0.3-3. Thresholds to identify expressed genes from background noise were then calculated as the median of the maxima of the negative control probe measurements for each cell type.

### Selection of microarray probesets for comparison between platforms

Microarray probesets mapping to genes measured by the nCounter Analysis System were identified by Affymetrix GeneChip Human Gene ST Arrays Probeset Annotations Release 33.2. Where multiple probesets were available for a given gene, the probeset with the best target region overlap with the nCounter target region was chosen. Where all probesets were equivalent in overlap, one was chosen at random (see Additional file 5 for probeset mappings).

### Correlation between platforms

nCounter data was log-transformed and normalized to either a single, or to the mean of two, log-transformed control gene measurements. Where indicated, microarray expression values were similarly normalized to single or multiple control gene expression values. The two platforms were compared by Pearson correlation of each gene across samples.

### Log-ratio accuracy and precision analysis

Signal detection slopes were calculated by taking the slope of a linear model fit to log-transformed microarray expression values versus log-transformed, control-gene-normalized nCounter measurements. Although spline-fitted curves are often used for similar spike-in experiments to allow for expression-level dependencies, expression of each gene is generally contained within a smaller range than spike-in controls, and therefore we found it appropriate to use linear regression for each individual gene. For this analysis, each nCounter dataset was normalized to two cell-type-specific control genes, while microarray datasets were not normalized to control genes. Noise in unexpressed microarray probesets was examined by measuring the standard deviation of all possible microarray log-ratios between pairs of samples for each gene in one of two sets: 1) all genes called unexpressed by the nCounter; and 2) all genes called invariant across samples by the nCounter (log-transformed, control-gene-normalized nCounter variance < 0.1) but strictly unsaturated on the microarray (microarray expression value median < 11, see Additional file 2).

## Results

### Microarray expression value level and variance indicate transcript presence and correlation with nCounter measurements

We compared a previously acquired single-color microarray dataset of 312 samples from 9 batches, containing multiple cell types and diagnoses (see Additional file 1), with nCounter data from 47 of these same RNA samples, acquired in 6 nCounter Analysis System runs over 8 days (see Additional file 6). An additional 7 RNA samples were measured by nCounter for inter-run comparisons. The nCounter panel was designed to detect transcripts of 65 genes, including cell-type-specific control genes, spanning a wide range of expression levels and variances (see Additional file 5). Two nCounter probesets were excluded: one for poor predicted hybridization specificity, and one based on hybridization failure. nCounter data was processed as described in Methods, using spiked positive and negative control probes to correct for hybridization efficiency and determine count thresholds for unexpressed genes, respectively. Technical replicates of the same sample on different nCounter runs were highly correlated (see Additional file 4), and variation between samples of different diagnoses was greater than that between samples of the same diagnosis in different nCounter runs (see Additional file 4), implying very little inter-run variability. Reduced inter-run correlations in CD14+ monocyte (CD14) samples were due to one outlier (see Additional file 4), which was not used in subsequent nCounter versus microarray comparisons. Based on this analysis, we considered nCounter run-effects negligible and processed all samples for each cell type together, disregarding run membership. As the nCounter Analysis System experiment measured a small number of genes, it was not possible to normalize across samples by fitting expression measures to a common distribution [26]. Instead, nCounter transcript counts were normalized to selected control genes (see Additional file 3), as described in Methods.

Because cell type greatly influences expression patterns [28], and because our microarray datasets consisted of cell-type-specific batches that confound effects from these two variables, each cell type was treated as a separate dataset for processing. Microarray datasets were preprocessed by RMA and, where applicable, corrected for batch effects by ComBat, as described in Methods. The resulting log-transformed, standardized microarray values are referred to as “microarray expression values” throughout this manuscript.

**Figure 1:**
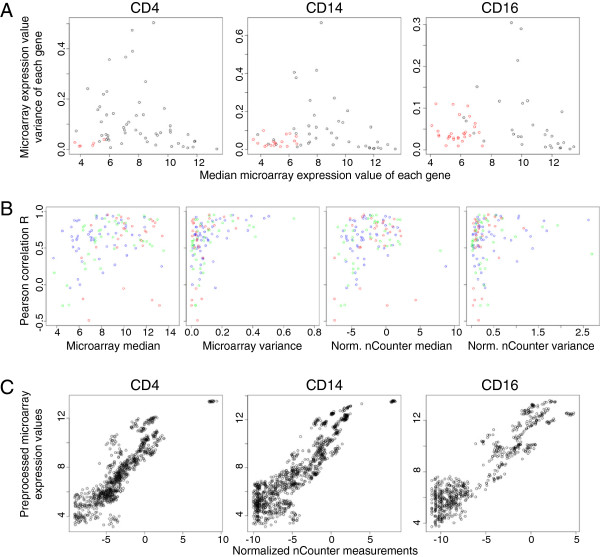
**Median and variance properties of expressed versus unexpressed genes and interplatform correlation. A)** Genes are plotted by cell-type-specific RMA + ComBat- (CD4 and CD14) or RMA-preprocessed (CD16) microarray expression value median and variance. Red indicates genes called unexpressed by nCounter measurement. **B)** nCounter datasets were normalized to two cell-type-specific control genes each, log-transformed and compared to RMA + ComBat- (CD4 and CD14) or RMA-preprocessed (CD16) microarray data without control-gene normalization. Plots show Pearson correlation of each expressed gene versus the median or variance of its microarray expression values or control-gene-normalized, log-transformed nCounter measurements: blue = CD4, green = CD14, red = CD16 datasets. **C)** RMA + ComBat- (CD4 and CD14) or RMA-preprocessed (CD16) microarray data plotted versus nCounter measurements normalized to two cell-type-specific control genes.

With nCounter thresholds determined from negative control probe counts, we identified expressed and unexpressed genes in each cell type. Unexpressed genes were characterized by both low expression and low variance on the microarray (Figure 1A). It is important to note that many genes identified as expressed by the nCounter analysis system had microarray expression values lower than those identified as unexpressed, confirming on a new platform the observation made by Irizarry and colleagues [35, 36] that probe-effects prevent strict, experiment-wide thresholding of expressed genes. Introducing a second variance threshold might improve identification of unexpressed genes, but a larger study examining more genes would be required to develop such a rule.

We examined the correlation of each expressed gene across samples between the microarray and nCounter platforms, varying the choice of control genes for nCounter data, the use or absence of control gene normalization for preprocessed microarray data, and the use or absence of microarray batch correction (Additional files 7 and 8). Choice of nCounter control genes changed correlation results slightly, with multiple control genes generally performing better than single control genes, as previously suggested for other technologies [37]. The poorer performance of CD4+ T cell (CD4) samples normalized to PIAS1 suggests that this gene was not as invariant as indicated by its microarray expression values, perhaps due to microarray saturation. Control-gene-normalizing the microarray data generally improved correlation, but the moderate extent of this improvement indicates acceptable robustness of the control genes used.

CD4 and CD14 RNA samples were run on multiple microarray batches, leaving microarray datasets potentially confounded by technical artefacts. Although principal component analyses of the entire datasets before batch correction demonstrated obvious batch effects (see Additional file 9), batch correction had very little effect on most genes examined in this study (see Additional files 7, 8, 9). In fact, correlation between microarray and nCounter measurements remained globally unchanged with batch correction of the CD4 and CD14 microarray datasets. Direct comparison of batch-corrected and non-batch-corrected data (see Additional file 9) indicates that this correction dramatically improved correlation of several genes in the CD14 dataset while the median gene correlation decreased slightly, possibly due to a reduction in covariance that would be predicted if batch correction reduced microarray variance in comparison to noise levels. These gene-specific effects highlight the importance of using batch-correction algorithms, such as ComBat, that can normalize at the gene level even with many covariate groups spread across batches.Examination of the inter-platform correlation coefficient for each expressed gene versus either its median expression level or variance (Figure 1B) demonstrated that high variance generally corresponded with good correlation. Indeed, Pearson correlation relies on the covariance of two variables, inherently requiring variation of both, but it is interesting to note that many low-variance genes also exhibited good inter-platform correlation. This empirically indicates high precision of microarray measurements, allowing successful identification of true transcript variation over platform-specific noise for many genes, even with low inter-sample variation. In comparisons of inter-platform correlation with median expression, genes with very low and very high expression generally had poorer correlation. Directly plotting microarray expression values against their corresponding control-gene-normalized nCounter measurements revealed large variability at the low end of expression and flattening of microarray values at the high end (Figure 1C). Taken together, these data suggest that low expression measurements on the microarray may have been obscured by background noise while high measurements were likely saturated.

### Microarray signal detection accuracy depends on expression level and is dataset-specific

**Figure 2:**
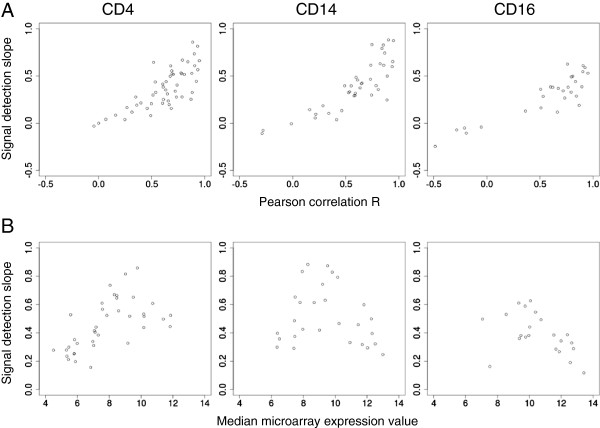
**Signal detection accuracy of microarray gene expression values. A)** Signal detection slopes of RMA + ComBat- (CD4 and CD14) or RMA-preprocessed (CD16) microarray expression values for all expressed genes are plotted against their inter-platform correlation coefficient. **B)** Signal detection slopes of RMA + ComBat- (CD4 and CD14) or RMA-preprocessed (CD16) microarray expression values for all genes with correlated nCounter and microarray measures (Pearson R > 0.5) are plotted against median microarray values.

One metric used for determining microarray measurement accuracy is the “signal detection slope” [5], or the slope of linear regression relating measured microarray expression values to log-transformed, known input transcript concentrations. A slope of one indicates that the microarray accurately reflects the input. To examine microarray accuracy using real experimental data, we calculated the signal detection slope of microarray expression values versus log-transformed, control-gene-normalized nCounter measurements by fitting linear regressions to the paired platform measurements of each gene. Figure 2A shows signal detection slope plotted against correlation coefficient. Because both correlation coefficients and signal detection slopes were determined by comparison of the same measurements, genes with low correlation also showed low signal detection. However, genes with high correlation did not necessarily have high signal detection, instead exhibiting a much wider range of signal detection slopes. In order to better understand this spread, we filtered for genes with good inter-platform correlation (Pearson’s correlation coefficient > 0.5) and plotted these signal detection slopes versus the median microarray expression value (Figure 2B). Signal detection accuracy was globally reduced (approximately 0.5), with slopes particularly dampened in genes with high (e.g. high end of CD16+ neutrophil, referred to as CD16, dataset) and low (e.g. low end of CD4 dataset) microarray expression values. Even within the same expression level ranges, signal detection varied by dataset, indicating variable effects of independent preprocessing and/or tissue type on accuracy reduction. Batch correction slightly reduced signal detection in general, again possibly due to covariance reduction through decreasing microarray variance (see Additional file 10).

### Noise in microarray expression values is dataset-specific

**Figure 3:**
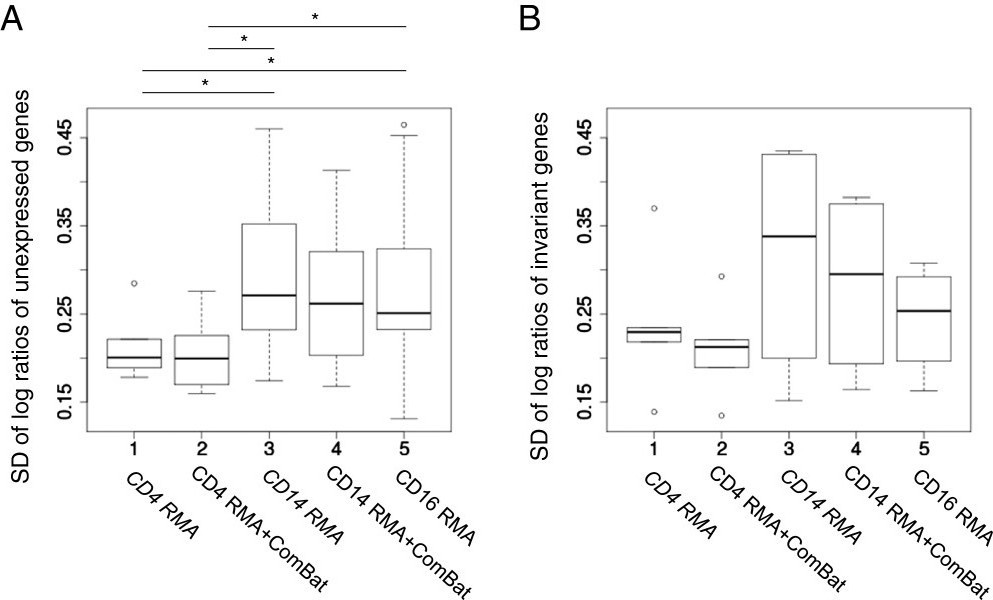
**Signal detection precision of microarray gene expression values. A)** Boxplots depict the standard deviations of log-ratios of all pairs of samples for unexpressed genes. Stars indicate Mann–Whitney test p-value < 0.05. **B)** As **A** for genes invariant by nCounter but unsaturated on the microarray.

As noted above, inter-platform correlation of low-variance genes demonstrated wide variability within and across datasets (Figure 1B), suggesting variable levels of noise in the microarray measurements. To examine microarray expression value precision in each dataset, we adapted a metric developed by McCall et al. [17] for spike-in data and examined the standard deviation of microarray log-ratios of unexpressed and invariant genes, as described in Methods. Noise of unexpressed genes varied between datasets (Figure 3A), indicating either a tissue-specific effect or an artefact of the preprocessing of each dataset. Batch correction slightly reduced this variation, suggesting improvements in precision. Comparison with microarray median expression values (see Additional file 11) demonstrated that noise amplitude of unexpressed genes remained largely independent of microarray expression values. The CD4 dataset had significantly less noise than the CD14 and CD16 datasets (Figure 3A), implying that comparisons of low variance gene expression values might be more reliable in this dataset. Indeed, this was observed in the generally improved inter-platform correlation coefficients of low-variance genes in the CD4 subset (Figure 1B, blue). Because noise in unexpressed genes only addresses precision at low microarray expression values and because our unexpressed gene sets were of different sizes, we also examined microarray precision in invariant genes over a wider range of expression. To this end, we used nCounter measurements to select genes with low variance across all samples disregarding diagnosis (normalized variance < 0.1, Figure 1B right panel) and strictly filtered for genes not saturated on the microarray. Examination of this noise metric across datasets (Figure 3B) revealed the same trends for batch correction and cell-type-specificity. Comparing precision of invariant gene measurements versus median expression values on the microarray (see Additional file 11) indicated that genes with very high expression tended to better precision. Although we had filtered strictly to avoid saturated microarray expression values, this precision trend, as well as the binned variance properties depicted in Additional file 2, suggests that saturation or compression artefacts of RMA preprocessing [6] may begin to reduce inter-sample variation at even lower expression values.

## Discussion

While we are not the first to ask questions about microarray interpretation of gene expression and log-ratios, our approach is unique in its use of a count-based technology to examine diverse biological samples. This has enabled us to examine the properties of microarray data representative of real experimental questions and to present the new observation of tissue-specific noise.

Through examination of inter-platform correlation and expressed versus unexpressed gene properties, we have demonstrated that variance measurement may aid in the determination of whether or not a gene is expressed, particularly at low microarray expression values. Irizarry and colleagues have clearly shown that a low microarray expression value does not necessarily predict an unexpressed gene [35, 36]. The small number of genes tested in the present study would suggest that simultaneous thresholds for microarray gene expression level and variance might perform better than expression level thresholds alone to identify present versus absent transcripts (Figure 1A), but nCounter measurement of a larger gene set would be necessary to develop such a method. Indeed present/absent calls, expression thresholds and, most popularly, variance filters [38] are frequently used in downstream microarray analyses to reduce multiple testing and improve power for discovery (as compared in [39]). Our demonstration that unexpressed genes generally have low microarray expression values and variances provides empirical justification particularly for using the intersection of such filters to remove uninformative probesets.

Our comparison of log-fold-changes measured by microarray and nCounter platforms has revealed that signal detection accuracy of the microarray varies dramatically by expression level. Similar to previous studies with constructed datasets [5, 6, 17, 18, 21], our data show global compression most extreme at very high and low gene expression values. Importantly, we noted that signal detection slope compression also varies by dataset, indicating that effect sizes measured by log-fold changes are not necessarily comparable between datasets preprocessed separately. Thus, comparing the magnitudes of expression changes in genes expressed at different levels or genes in different datasets requires knowledge of their individual signal detection slopes.

For precision estimation, we examined noise of genes defined as unexpressed or invariant by nCounter measurements. The RMA algorithm stabilizes variance with respect to expression level [8], and thus noise patterns from probesets detecting unexpressed genes should reflect noise across the whole microarray. Accordingly, we found the same patterns of precision estimates in unexpressed genes as in invariant genes across a wider range of expression levels. We noted that noise appeared strongly tissue-dependent, with less in the CD4 than the CD14 or CD16 datasets. Binned mean expression versus variance plots (see Additional file 2) support this pattern of tissue-specific precision, showing a high-variance peak at very low expression levels in the CD14 and CD16 datasets (likely representing unexpressed gene noise) that is nearly absent in the CD4 dataset. Because the same RNA was used for both the nCounter and microarray measurements, dataset-dependent noise differences can only be due to properties intrinsic to the frozen RNA samples. Transcriptomes differ by tissue (see Additional file 12 and [36]), and thus one explanation for differing noise levels is that levels of cross-hybridizing nucleotides also vary by cell type. We previously showed that RNA transcript profiles of cells from the myeloid lineage (CD14+ monocytes and CD16+ neutrophils) change dramatically if blood is left several hours before processing [28]. Although we found no evidence of large-scale loss of RNA integrity, it is possible that even during rapid blood processing, RNA from myeloid cells suffers slightly more degradation, a factor likely to confound microarray more than the nCounter measurements [24]. Another plausible explanation is cell-type-specific contamination with genomic DNA, particularly in the CD16+ neutrophil subset, which has comparatively less RNA per cell [28] and thus a higher ratio of genomic DNA to RNA. Genomic DNA would likely be problematic in the transcriptional step of microarray sample preparation [2] but not the amplification-free nCounter procedure [24]. Regardless of the source of this dataset-specific noise, such effects are important to remember for cross-tissue studies, such as the Gene Expression Barcode [35, 36], where probes reflecting signal in one tissue type may be conflated by noise in another. Our precision results are based on examination of a limited number of genes in three different leukocyte subsets, and future studies of more genes in additional tissue types will begin to shed light on the origin and extent of this dataset-specific noise.

## Conclusions

This analysis of gene expression microarray measurements versus transcript count ratios highlights three aspects of microarray data directly relevant to users of the technology. First, inter-sample variance may indicate transcript presence in genes with low microarray expression values. Second, signal detection accuracy depends strongly on expression level, even in datasets of diverse biological samples with variable background and small gene expression ranges. Third, precision is dataset-specific, and therefore power to detect subtle biological differences may differ between tissues even when measured on the same microarray platform. Without careful consideration of these biases and confirmatory measurements by a second technology, microarray platform discoveries may be missed or misinterpreted.

## Availability of supporting data

The data sets supporting the results of this article are available in the ArrayExpress repository: https://www.ebi.ac.uk/arrayexpress/experiments/E-MTAB-2452/.

## Electronic supplementary material

Additional file 1:: **Preprocessing samples.** Microarray samples used for preprocessing: Breakdown of preprocessed microarray batches including biological covariates. (PDF 59 KB)

Additional file 2:: **Mean v variance.** Binned mean and variance characteristics of microarray datasets: For each gene, a microarray expression value mean and mean within-batch variance was calculated. Genes were then binned by expression value means, and statistics were averaged to achieve an average mean expression value and average mean within-batch-variance for each bin. These two values are plotted. (PDF 64 KB)

Additional file 3:: **Control genes.** Microarray properties of nCounter control genes: Details of each cell-type-specific control gene used. (PDF 40 KB)

Additional file 4:: **nCounter run comparison.** Examination of inter-run technical effects of nCounter data: A) Log-transformed raw nCounter counts for technical replicates of the same sample are plotted with Pearson correlation indicated. All genes determined to be globally expressed in CD4 samples are included. B) Boxplots depict Pearson correlations between log-transformed raw nCounter counts for samples of the same and different diagnoses in the same and in different nCounter runs. All genes determined to be globally expressed in the designated cell types are included. In the bottom panel, the outlying CD14 sample has been removed. C) Inter-sample Pearson correlation coefficients of log-transformed raw nCounter counts between all CD14 samples. Red star indicates outlier. (PDF 131 KB)

Additional file 5:: **nCounter probes.** nCounter probe details and mapped Affymetrix Hugene 1.1 ST array probesets: nCounter probe design schemes, isoform coverage, and microarray probeset mappings are tabulated. (PDF 96 KB)

Additional file 6:: **nCounter samples.** Sample composition: Details are provided for samples run on the nCounter analysis system. (PDF 91 KB)

Additional file 7:: **Correlation comparison.** Effects of microarray and nCounter processing on inter-platform correlation: Cell-type-specific nCounter datasets were normalized to the indicated control genes and log-transformed. Microarray data were preprocessed by RMA and then batch normalized through ComBat and/or normalized to control genes where indicated. Boxplots show Pearson correlation. (PDF 76 KB)

Additional file 8:: **Correlation comparison table.** Effects of microarray and nCounter processing on inter-platform correlation: Table summarizes inter-platform correlation of datasets using different processing and normalization procedures. (PDF 66 KB)

Additional file 9:: **Microarray batch effects.** Batch effects in microarray datasets: A) Samples from full CD4 and CD14 microarray datasets are plotted by first and second principle components before and after ComBat batch correction. Color indicates batch membership. B) Pearson correlation of expressed genes across samples in nCounter versus RMA-preprocessed microarray datasets was subtracted from the same correlation in nCounter versus RMA-preprocessed and ComBat-corrected microarray datasets. Boxplots depict these differences in CD4 and CD14 datasets to indicate the effect of batch correction on gene-based platform correlation. (PDF 77 KB)

Additional file 10:: **Batch correction and accuracy.** Effect of batch correction on signal detection accuracy: A) Signal detection slope is plotted versus inter-platform correlation as in Figure 2A: blue = RMA- and red = RMA + ComBat-preprocessed microarray expression values. B) Signal detection slope of expressed genes across samples in nCounter versus RMA-preprocessed microarray datasets was subtracted from the same signal detection slope in nCounter versus RMA-preprocessed and ComBat-corrected microarray datasets. Boxplots depict these differences in CD4 and CD14 datasets to indicate the effect of batch correction on signal detection accuracy. (PDF 66 KB)

Additional file 11:: **Noise v expression value.** Comparison of noise versus microarray expression value. A) For each unexpressed gene, the standard deviation of log-ratios of all pairs of samples from RMA + ComBat- (CD4 and CD14) or RMA-preprocessed (CD16) microarray data is plotted versus the gene’s median microarray expression value. B) As (A) for invariant genes. (PDF 81 KB)

Additional file 12:: **Mean expression histograms.** Mean expression profiles: Histograms depict mean RMA + ComBat- (CD4 and CD14) or RMA-preprocessed (CD16) microarray expression values from full microarray datasets. (PDF 65 KB)

## Abbreviations

RMA: Robust multi-array average
qPCR: quantitative polymerase chain reaction
AAV: Anti-neutrophil-cytoplasmic-antibody-associated vasculitis
IBD: Inflammatory bowel disease
CD: Crohn’s disease
UC: Ulcerative colitis
GPA: Granulomatosis with polyangiitis
MPA: Microscopic polyangiitis.
