# Supplementary material for: Comparison of gene expression microarray data with count-based RNA measurements informs microarray interpretation
Source: BMC Genomics. 2014 Aug 4;15(1):649. doi: 10.1186/1471-2164-15-649 (PMC4143561; doi:10.1186/1471-2164-15-649)
Supplement: Supplementary file 1 — Additional file 1:: Preprocessing samples. Microarray samples used for preprocessing: Breakdown of preprocessed microarray batches including biological covariates. (PDF 59 KB) [file 12864_2014_6367_MOESM1_ESM.pdf]

Additional file 1: Microarray samples used for pre-processing.

Cell Type	Batch	# Samples used for Normalization	Diagnosis Breakdown (CD, UC, GPA, MPA, HC)	M:F Ratio	Median Age
CD4	52	34	6/28/0/0/0	0.79	41
CD4	70	24	0/0/9/9/6	1.00	61.5
CD4	71	23	6/3/0/5/9	0.77	30
CD4	72	18	11/1/0/0/6	0.29	38
CD14	58	41	6/29/0/0/6	0.86	44
CD14	62	25	0/0/11/14/0	0.79	60
CD14	64	10	0/0/7/0/3	0.43	50
CD14	67	18	0/0/9/9/0	1.25	70
CD14	71	30	9/5/4/3/9	1.00	35.5
CD16	60	89	39/31/0/0/19	0.65	36

Diagnosis breakdown: CD = Crohn's disease, UC = ulcerative colitis, GPA = granulomatosis with polyangiitis, MPA = microscopic polyangiitis, HC = health control.
