# Supplementary material for: Comparison of gene expression microarray data with count-based RNA measurements informs microarray interpretation
Source: BMC Genomics. 2014 Aug 4;15(1):649. doi: 10.1186/1471-2164-15-649 (PMC4143561; doi:10.1186/1471-2164-15-649)
Supplement: Supplementary file 2 — Additional file 2:: Mean v variance. Binned mean and variance characteristics of microarray datasets: For each gene, a microarray expression value mean and mean within-batch variance was calculated. Genes were then binned by expression value means, and statistics were averaged to achieve an average mean expression value and average mean within-batch-variance for each bin. These two values are plotted. (PDF 64 KB) [file 12864_2014_6367_MOESM2_ESM.pdf]

Additional File 2

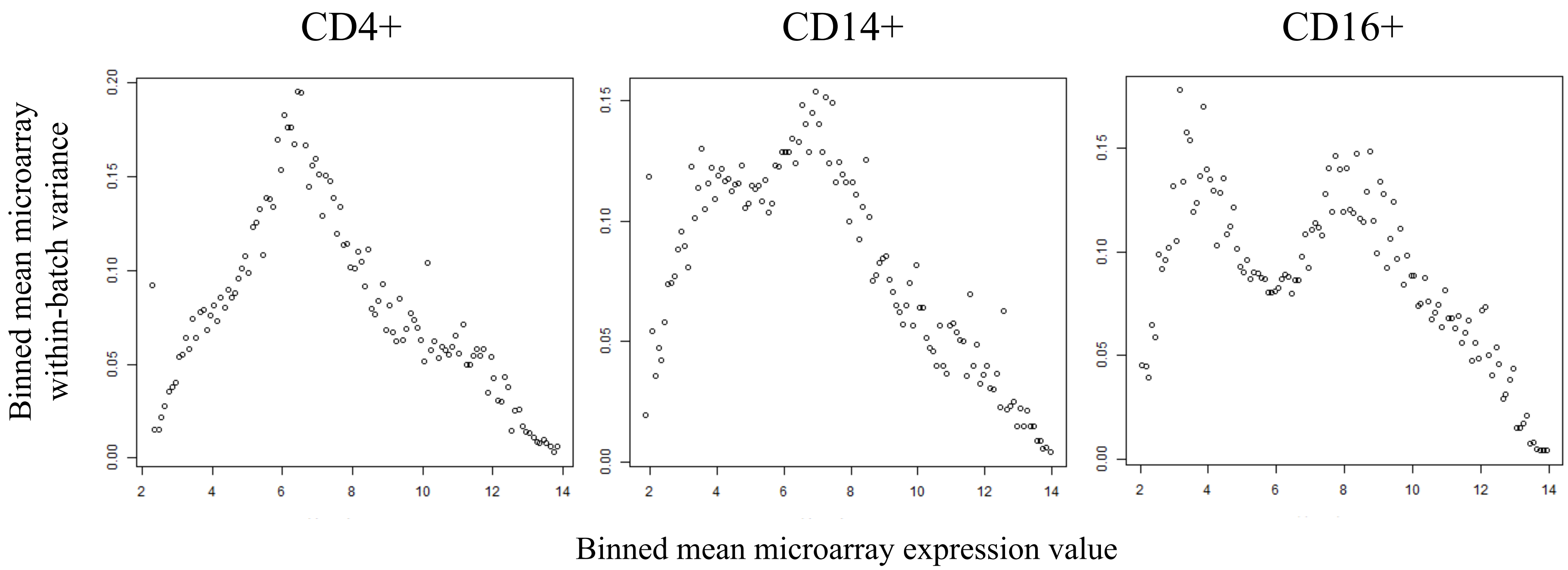

Binned mean and variance characteristics of microarray datasets. For each gene, a microarray expression value mean and mean within-batch variance was calculated. Genes were then binned by expression value means, and statistics averaged to achieve an average mean expression value and average mean within-batch-variance for each bin. These two values are plotted.
