# Supplementary material for: Comparison of gene expression microarray data with count-based RNA measurements informs microarray interpretation
Source: BMC Genomics. 2014 Aug 4;15(1):649. doi: 10.1186/1471-2164-15-649 (PMC4143561; doi:10.1186/1471-2164-15-649)
Supplement: Supplementary file 3 — Additional file 3:: Control genes. Microarray properties of nCounter control genes: Details of each cell-type-specific control gene used. (PDF 40 KB) [file 12864_2014_6367_MOESM3_ESM.pdf]

Additional File 3: Microarray properties of nCounter control genes

Control gene	Cell type	Mean microarray expression value	Mean of batch-specific micrarray variance	Rank of variance after mean filter
CNOT1	CD4	11.40	0.0118	163
	CD14	11.74	0.0060	20
	CD16	11.60	0.0061	14
PIAS1	CD4	11.74	0.0072	9
PCBP1	CD14	11.99	0.0053	7
CELF2	CD16	11.97	0.0072	36
