# Supplementary material for: Comparison of gene expression microarray data with count-based RNA measurements informs microarray interpretation
Source: BMC Genomics. 2014 Aug 4;15(1):649. doi: 10.1186/1471-2164-15-649 (PMC4143561; doi:10.1186/1471-2164-15-649)
Supplement: Supplementary file 4 — Additional file 4:: nCounter run comparison. Examination of inter-run technical effects of nCounter data: A) Log-transformed raw nCounter counts for technical replicates of the same sample are plotted with Pearson correlation indicated. All genes determined to be globally expressed in CD4 samples are included. B) Boxplots depict Pearson correlations between log-transformed raw nCounter counts for samples of the same and different diagnoses in the same and in different nCounter runs. All genes determined to be globally expressed in the designated cell types are included. In the bottom panel, the outlying CD14 sample has been removed. C) Inter-sample Pearson correlation coefficients of log-transformed raw nCounter counts between all CD14 samples. Red star indicates outlier. (PDF 131 KB) [file 12864_2014_6367_MOESM4_ESM.pdf]

Additional File 4

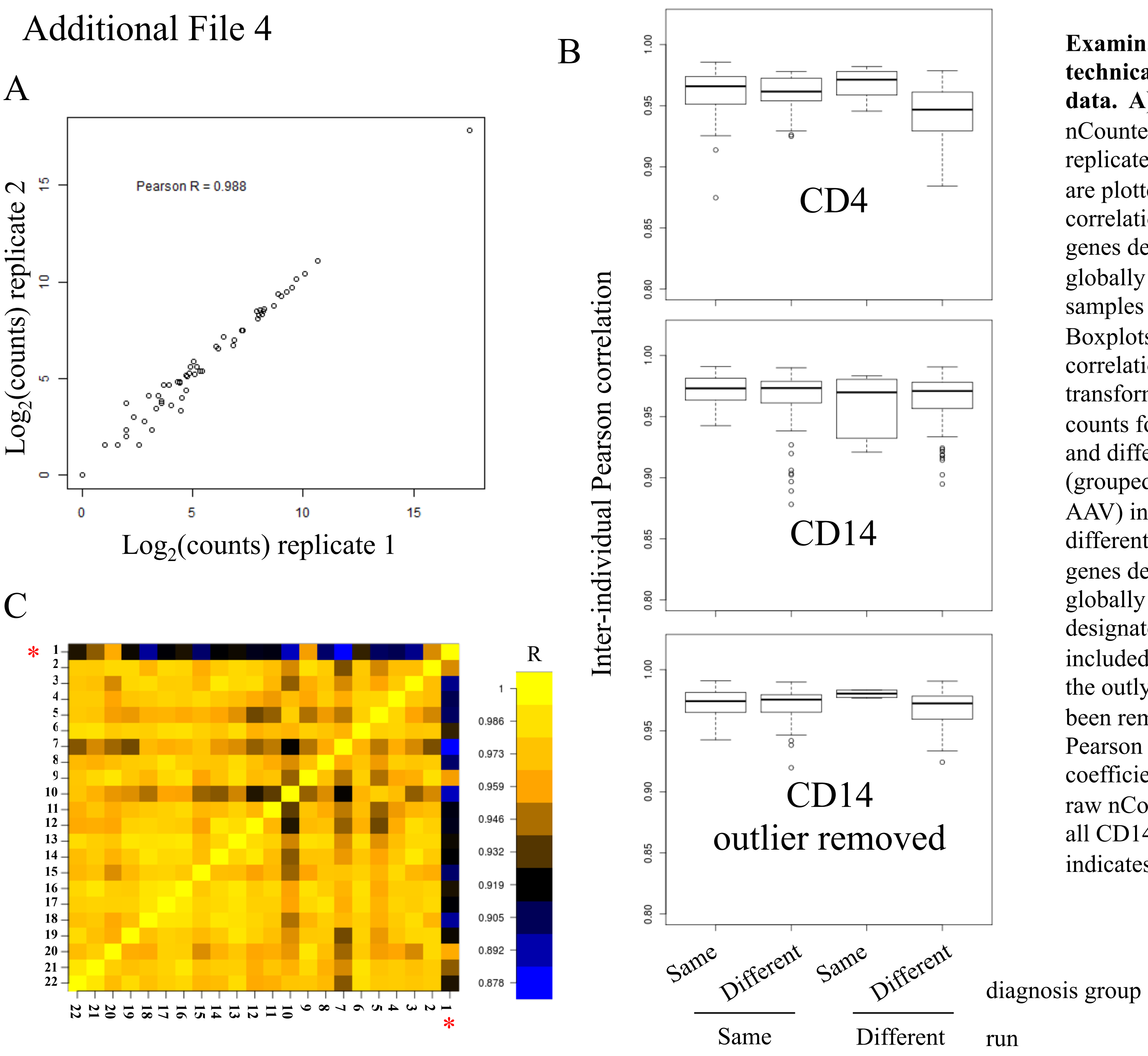

Examination of inter-run technical effects of nCounter data. **A**) Log-transformed raw nCounter counts for technical replicates of the same sample are plotted with Pearson correlation indicated. All genes determined to be globally expressed in CD4 samples are included. **B**) Boxplots depict Pearson correlations between log-transformed raw nCounter counts for samples of the same and different diagnoses (grouped as Control, IBD, AAV) in the same and in different nCounter runs. All genes determined to be globally expressed in the designated cell types are included. In the bottom panel, the outlying CD14 sample has been removed. **C**) Inter-sample Pearson correlation coefficients of log-transformed raw nCounter counts between all CD14 samples. Red star indicates outlier.
