# Supplementary material for: Comparison of gene expression microarray data with count-based RNA measurements informs microarray interpretation
Source: BMC Genomics. 2014 Aug 4;15(1):649. doi: 10.1186/1471-2164-15-649 (PMC4143561; doi:10.1186/1471-2164-15-649)
Supplement: Supplementary file 5 — Additional file 5:: nCounter probes. nCounter probe details and mapped Affymetrix Hugene 1.1 ST array probesets: nCounter probe design schemes, isoform coverage, and microarray probeset mappings are tabulated. (PDF 96 KB) [file 12864_2014_6367_MOESM5_ESM.pdf]

Additional File 5: nCounter probe details and mapped Affymetrix Hugene 1.1 ST array probesets

Gene Symbol	Accession of target transcript (RefSeq)	Target region of target transcript	Isoforms hit by probe (RefSeq)	Isoforms not hit by probe	Mapped Human Gene 1.1 ST probeset or why excluded
ARPC4	NM_005718.4	970-1070	NM_005718; NM_001198780; NM_001024960; NM_001024959		No probesets map to only this gene
BHLHE40	NM_003670.1	560-660	NM_003670		8077441
C15orf39	NM_015492.4	2135-2235	NM_015492		7984952
CCNY	NM_145012.4	385-485	NM_145012; NM_181698		7927008
CD27	NM_001242.4	330-430	NM_001242		7953333
CD40	NM_001250.4	1265-1365	NM_001250; NM_152854		8063156
CD40LG	NM_000074.2	1225-1325	NM_000074		8170187
CD70	NM_001252.2	190-290	NM_001252		8033241
CELF2	NM_001025076.2	395-495	NM_001025076; NM_001083591; NM_006561; NM_001025077		7926127
CNOT1	NM_206999.1	405-505	NM_206999; NM_016284		8001693
EDA	NM_001399.4	2435-2535	NM_001399; NM_001005612; NM_001005609	NM_001005613; NM_001005610	8168062
EDA2R	NM_001199687.2	1195-1295	NM_001199687; NM_021783; NM_001242310		8173299
EDAR	NM_022336.1	2990-3090	NM_022336		8054451
EEF1A1	NM_001402.5	0-100	NM_001402		8158952
FAS	NM_152876.1	1740-1840	NM_152876; NR_028036; NR_028035; NR_028034; NR_028033; NM_152872; NM_152871; NM_000043		7929032
FASLG	NM_000639.1	625-725	NM_000639		7907430
HIST1H1B	NM_005322.2	659-759	NM_005322		8124527
HLA-DRB5	NM_002125.3	131-231	NM_002125		nCounter probe flagged for non-specific hybridization
IL23R	NM_144701.2	710-810	NM_144701		7902189
LTA	NM_001159740.1	880-980	NM_001159740; NM_000595		8179258
LTB	NM_002341.1	330-430	NM_002341; NM_009588		8124950
LTBR	NM_002342.1	1435-1535	NM_002342		7953321

NEO1	NM_002499.2	2430-2530	NM_002499; NM_001172623; NM_001172624		7984704
NGFR	NM_002507.3	888-988	NM_002507; XM_003315367; XM_001090039		8008201
PCBP1	NM_006196.3	1252-1352	NM_006196		8042515
PIAS1	NM_016166.1	651-751	NM_016166		7984453
RELT	NM_032871.3	1978-2078	NM_032871; NM_152222		7942439
RORC	NM_001001523.1	1350-1450	NM_001001523; NM_005060		7920082
RPLP0	NM_001002.3	20-120	NM_001002; NM_053275		nCounter probe failure
SLC4A10	NM_001178016.1	2365-2465	NM_001178016; NM_001178015; NM_022058		8045974
SPTSSB	NM_001040100.1	715-815	NM_001040100		8091799
TCF7L2	NM_030756.3	600-700	NM_030756; NM_001198531; NM_001198530; NM_001198529; NM_001198528; NM_001198527; NM_001198526; NM_001198525; NM_001146274; NM_001146286; NM_001146285; NM_001146284; NM_001146283		7930537
TNF	NM_000594.2	617-717	NM_000594		8118142
TNFRSF10A	NM_003844.2	950-1050	NM_003844		8149762
TNFRSF10B	NM_147187.1	1495-1595	NM_147187; NR_027140; NM_003842		8149733
TNFRSF10C	NM_003841.2	5-105	NM_003841		8145244
TNFRSF10D	NM_003840.3	2355-2455	NM_003840		8149749
TNFRSF11A	NM_003839.2	490-590	NM_003839		8021528
TNFRSF11B	NM_002546.2	1075-1175	NM_002546		8152512
TNFRSF12A	NM_016639.2	791-891	NM_016639		7992789
TNFRSF13B	NM_012452.2	160-260	NM_012452		8013061
TNFRSF13C	NM_052945.3	789-889	NM_052945		8076387
TNFRSF14	NM_003820.2	916-1016	NM_003820		7911754
TNFRSF17	NM_001192.2	635-735	NM_001192		7993267
TNFRSF18	NM_148901.1	302-402	NM_148901; NM_148902; NM_004195		7911403
TNFRSF19	NM_018647.3	1192-1292	NM_018647; NM_001204459; NM_001204458; NM_148957		7968015

TNFRSF1A	NM_001065.3	1202-1302	NM_001065		7960518
TNFRSF1B	NM_001066.2	2835-2935	NM_001066		7897877
TNFRSF21	NM_014452.3	735-835	NM_014452		8126839
TNFRSF25	NM_148970.1	57-157	NM_148970; NM_001039664; NM_148967; NM_148966; NM_148965; NM_003790		7912040
TNFRSF4	NM_003327.2	200-300	NM_003327		7911413
TNFRSF6B	NM_003823.2	315-415	NM_003823		No probesets map to only this gene
TNFRSF8	NM_001243.3	3355-3455	NM_001243; NM_152942		7897860
TNFRSF9	NM_001561.5	1847-1947	NM_001561		7912145
TNFSF10	NM_003810.2	115-215	NM_003810; NR_033994; NM_001190943; NM_001190942		8092169
TNFSF11	NM_003701.2	490-590	NM_003701; NM_033012		7968851
TNFSF12	NM_003809.2	812-912	NM_003809; NR_037146		No probesets map to only this gene
TNFSF13	NM_003808.3	810-910	NM_003808; NM_172087; NM_172088; NM_001198624; NM_001198623; NM_001198622		No probesets map to only this gene
TNFSF13B	NM_006573.4	1430-1530	NM_006573; NM_001145645		7969986
TNFSF14	NM_003807.2	270-370	NM_003807; NM_172014		8033248
TNFSF15	NM_005118.2	350-450	NM_005118; NM_001204344		8163618
TNFSF18	NM_005092.2	175-275	NM_005092		7922337
TNFSF4	NM_003326.2	545-645	NM_003326		7922343
TNFSF8	NM_001244.3	518-618	NM_001244; NM_001252290		8163629
TNFSF9	NM_003811.3	398-498	NM_003811		8025053
