# Supplementary material for: Comparison of gene expression microarray data with count-based RNA measurements informs microarray interpretation
Source: BMC Genomics. 2014 Aug 4;15(1):649. doi: 10.1186/1471-2164-15-649 (PMC4143561; doi:10.1186/1471-2164-15-649)
Supplement: Supplementary file 6 — Additional file 6:: nCounter samples. Sample composition: Details are provided for samples run on the nCounter analysis system. (PDF 91 KB) [file 12864_2014_6367_MOESM6_ESM.pdf]

Additional File 6: Sample composition

nCounter run	Cell Type	Diagnosis	Gender	Age	Microarray batch
A	CD4	CD	M	41	52
A	CD4	UC	F	23	52
A	CD4	UC	M	32	52
A	CD4	UC	F	85	52
A	CD4	UC	M	50	52
A and F (technical replicate)	CD4	Control	F	27	71
A	CD4	Control	F	36	71
A	CD4	CD	F	28	72
A	CD4	CD	M	48	72
A	CD4	CD	F	67	72
A	*CD4*	*Control*	*M*	*36*	*used only for nCounter run comparison*
A	*CD4*	*Control*	*M*	*56*	*used only for nCounter run comparison*
B	CD4	GPA	M	73	70
B	CD4	GPA	F	62	70
B	CD4	GPA	M	44	70
B	CD4	GPA	M	74	70
B	CD4	MPA	F	80	70
B	CD4	MPA	M	83	70
C	CD14	CD	F	19	58
C	CD14	CD	M	31	58
C	CD14	CD	F	28	58
C	CD14	UC	F	85	58
C	CD14	UC	M	28	58
C	CD14	UC	F	34	58
C	CD14	UC	M	50	58
C	CD14	UC	M	41	58
C	CD14	Control	F	36	64
C	CD14	Control	F	58	71
C	CD14	Control	F	25	71
C	*CD14*	*Control*	*F*	*29*	*used only for nCounter run comparison*
D	CD14	GPA	M	65	62
D	CD14	MPA	M	59	62
D	CD14	MPA	F	50	62
D	CD14	GPA	F	53	64
D	CD14	MPA	M	68	67
D	CD14	MPA	M	59	67
D	CD14	MPA	M	83	67

D	CD14	MPA	M	68	67
D	*CD14*	*Control*	*M*	*62*	*used only for nCounter run comparison*
D	*CD14*	*Control*	*M*	*31*	*used only for nCounter run comparison OUTLIER*
D	*CD4*	*Control*	*F*	*36*	*used only for nCounter run comparison*
D	*CD4*	*Control*	*M*	*31*	*used only for nCounter run comparison*
E	CD16	CD	F	67	60
E	CD16	CD	F	19	60
E	CD16	CD	F	23	60
E	CD16	CD	F	35	60
E	CD16	Control	F	29	60
E	CD16	Control	M	36	60
E	CD16	Control	F	24	60
E	CD16	Control	F	28	60
E	CD16	CD	M	30	60
E	CD16	UC	F	54	60
E	CD16	UC	F	51	60
E	CD16	UC	M	50	60

Italics indicate samples used only for nCounter run comparisons.
