# Supplementary material for: Comparison of gene expression microarray data with count-based RNA measurements informs microarray interpretation
Source: BMC Genomics. 2014 Aug 4;15(1):649. doi: 10.1186/1471-2164-15-649 (PMC4143561; doi:10.1186/1471-2164-15-649)
Supplement: Supplementary file 7 — Additional file 7:: Correlation comparison. Effects of microarray and nCounter processing on inter-platform correlation: Cell-type-specific nCounter datasets were normalized to the indicated control genes and log-transformed. Microarray data were preprocessed by RMA and then batch normalized through ComBat and/or normalized to control genes where indicated. Boxplots show Pearson correlation. (PDF 76 KB) [file 12864_2014_6367_MOESM7_ESM.pdf]

Additional File 7

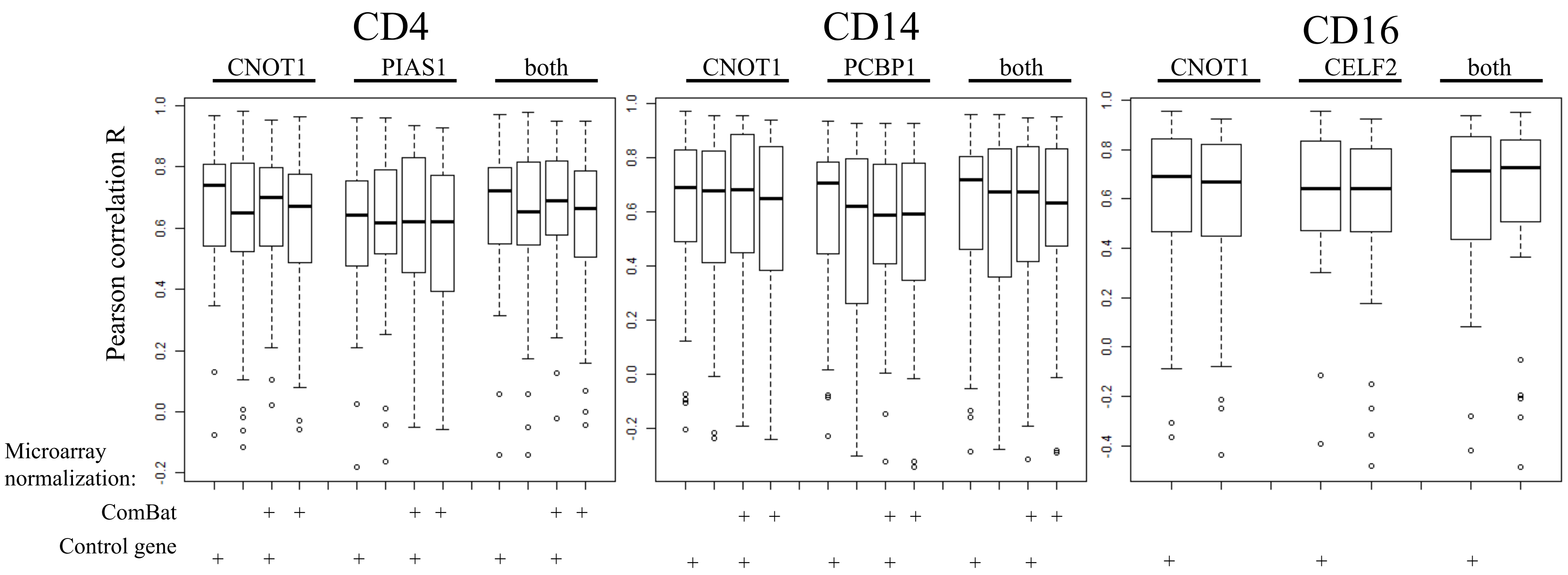

Effects of microarray and nCounter processing on inter-platform correlation. Cell-type-specific nCounter datasets were normalized to the indicated control genes and log-transformed. Microarray data was preprocessed by RMA and then batch normalized through ComBat and/or normalized to control genes where indicated. Boxplots show Pearson correlation.
