# Supplementary material for: Comparison of gene expression microarray data with count-based RNA measurements informs microarray interpretation
Source: BMC Genomics. 2014 Aug 4;15(1):649. doi: 10.1186/1471-2164-15-649 (PMC4143561; doi:10.1186/1471-2164-15-649)
Supplement: Supplementary file 8 — Additional file 8:: Correlation comparison table. Effects of microarray and nCounter processing on inter-platform correlation: Table summarizes inter-platform correlation of datasets using different processing and normalization procedures. (PDF 66 KB) [file 12864_2014_6367_MOESM8_ESM.pdf]

Additional File 8: Effects of microarray and nCounter processing on inter-platform correlation.

	Microarray: RMA + Control-gene norm		Microarray: RMA		Microarray: RMA+ComBat + Control-gene norm		Microarray: RMA+ComBat	
**CD4**	**Med**	**% > 0.5**	**Med**	**% > 0.5**	**Med**	**% > 0.5**	**Med**	**% > 0.5**
normalized to CNOT1	0.739	0.788	0.649	0.788	0.699	0.769	0.671	0.731
normalized to PIAS1	0.642	0.692	0.617	0.769	0.619	0.673	0.620	0.712
normalized to CNOT1 and PIAS1	0.722	0.824	0.654	0.784	0.689	0.804	0.664	0.745
**CD14**	**Med**	**% > 0.5**	**Med**	**% > 0.5**	**Med**	**% > 0.5**	**Med**	**% > 0.5**
normalized to CNOT1	0.690	0.750	0.676	0.700	0.683	0.750	0.650	0.700
normalized to PCBP1	0.705	0.675	0.622	0.575	0.588	0.625	0.590	0.575
normalized to CNOT1 and PCBP1	0.720	0.692	0.673	0.692	0.675	0.718	0.635	0.718
**CD16**	**Med**	**% > 0.5**	**Med**	**% > 0.5**				
normalized to CNOT1	0.692	0.655	0.666	0.621				
normalized to CELF2	0.643	0.690	0.641	0.690				
normalized to CNOT1 and CELF2	0.715	0.679	0.725	0.786				

Med = median Pearson correlation coefficient; % > 0.5 = percent of genes with Pearson correlation coefficient greater than 0.5.
