# Supplementary material for: Comparison of gene expression microarray data with count-based RNA measurements informs microarray interpretation
Source: BMC Genomics. 2014 Aug 4;15(1):649. doi: 10.1186/1471-2164-15-649 (PMC4143561; doi:10.1186/1471-2164-15-649)
Supplement: Supplementary file 9 — Additional file 9:: Microarray batch effects. Batch effects in microarray datasets: A) Samples from full CD4 and CD14 microarray datasets are plotted by first and second principle components before and after ComBat batch correction. Color indicates batch membership. B) Pearson correlation of expressed genes across samples in nCounter versus RMA-preprocessed microarray datasets was subtracted from the same correlation in nCounter versus RMA-preprocessed and ComBat-corrected microarray datasets. Boxplots depict these differences in CD4 and CD14 datasets to indicate the effect of batch correction on gene-based platform correlation. (PDF 77 KB) [file 12864_2014_6367_MOESM9_ESM.pdf]

Additional File 9

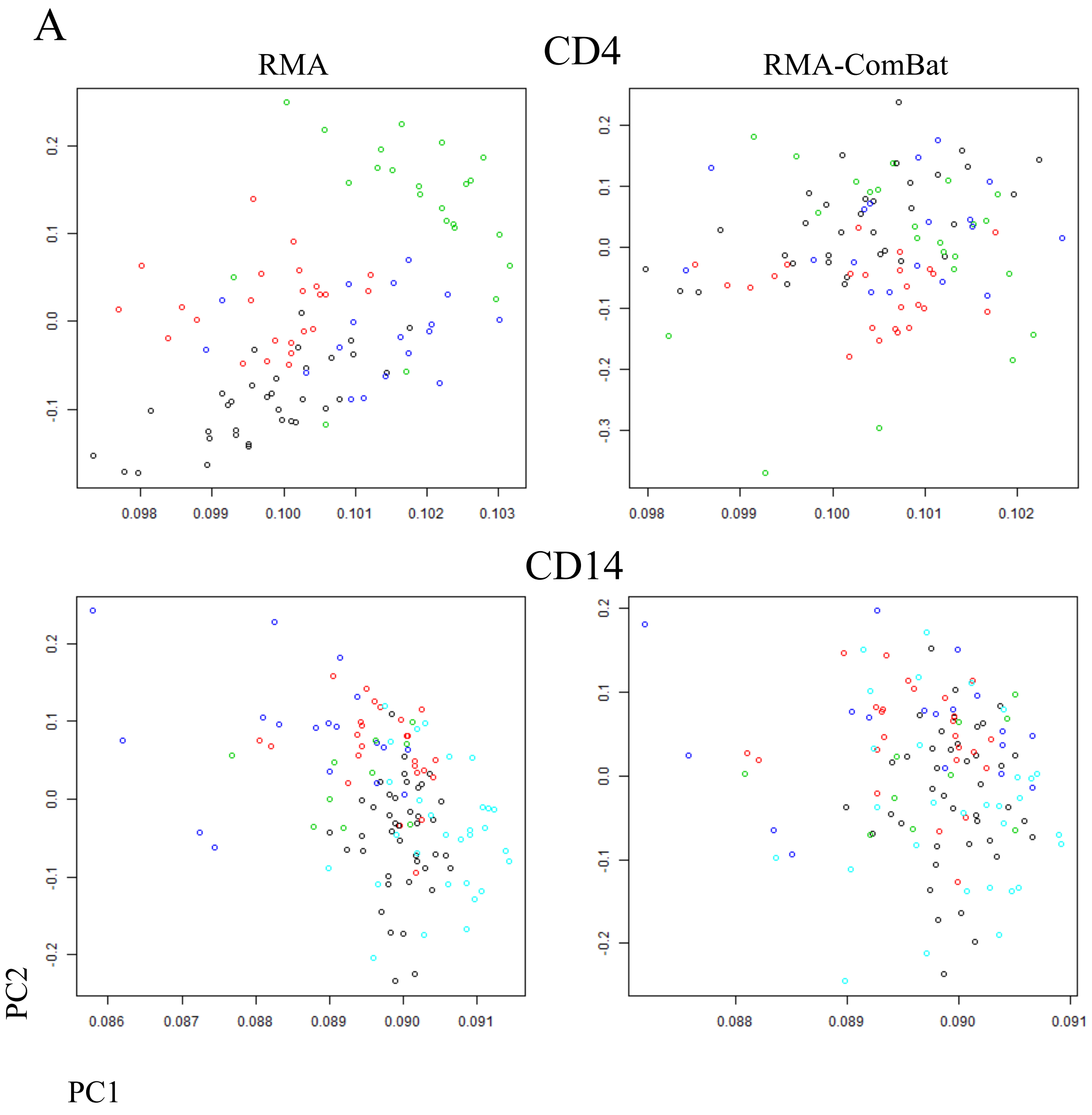

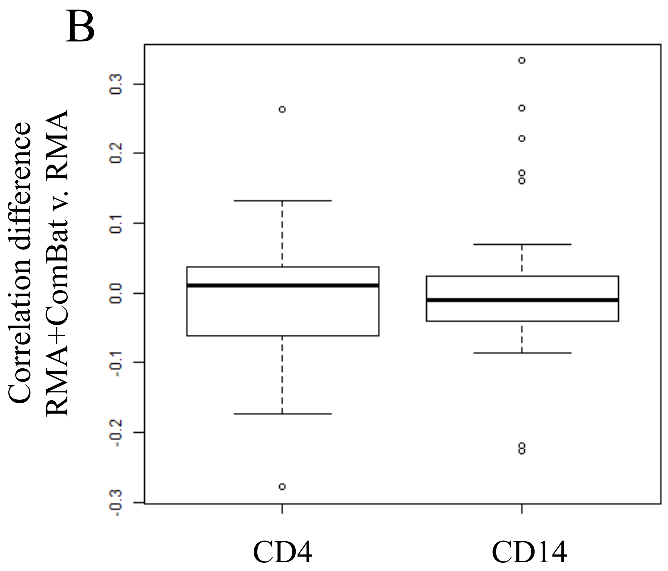

Batch effects in microarray datasets. A) Samples from full CD4 and CD14 microarray datasets are plotted by first and second principle components before and after ComBat batch correction. Color indicates batch membership. **B**) Pearson correlation of expressed genes across samples in nCounter versus RMA-preprocessed microarray datasets was subtracted from the same correlation in nCounter versus RMA-preprocessed and ComBat-corrected microarray datasets. Boxplots depict these differences in CD4 and CD14 datasets to indicate the effect of batch correction on gene-based platform correlation.
