# Supplementary material for: Comparison of gene expression microarray data with count-based RNA measurements informs microarray interpretation
Source: BMC Genomics. 2014 Aug 4;15(1):649. doi: 10.1186/1471-2164-15-649 (PMC4143561; doi:10.1186/1471-2164-15-649)
Supplement: Supplementary file 10 — Additional file 10:: Batch correction and accuracy. Effect of batch correction on signal detection accuracy: A) Signal detection slope is plotted versus inter-platform correlation as in Figure 2A: blue = RMA- and red = RMA + ComBat-preprocessed microarray expression values. B) Signal detection slope of expressed genes across samples in nCounter versus RMA-preprocessed microarray datasets was subtracted from the same signal detection slope in nCounter versus RMA-preprocessed and ComBat-corrected microarray datasets. Boxplots depict these differences in CD4 and CD14 datasets to indicate the effect of batch correction on signal detection accuracy. (PDF 66 KB) [file 12864_2014_6367_MOESM10_ESM.pdf]

Additional File 10

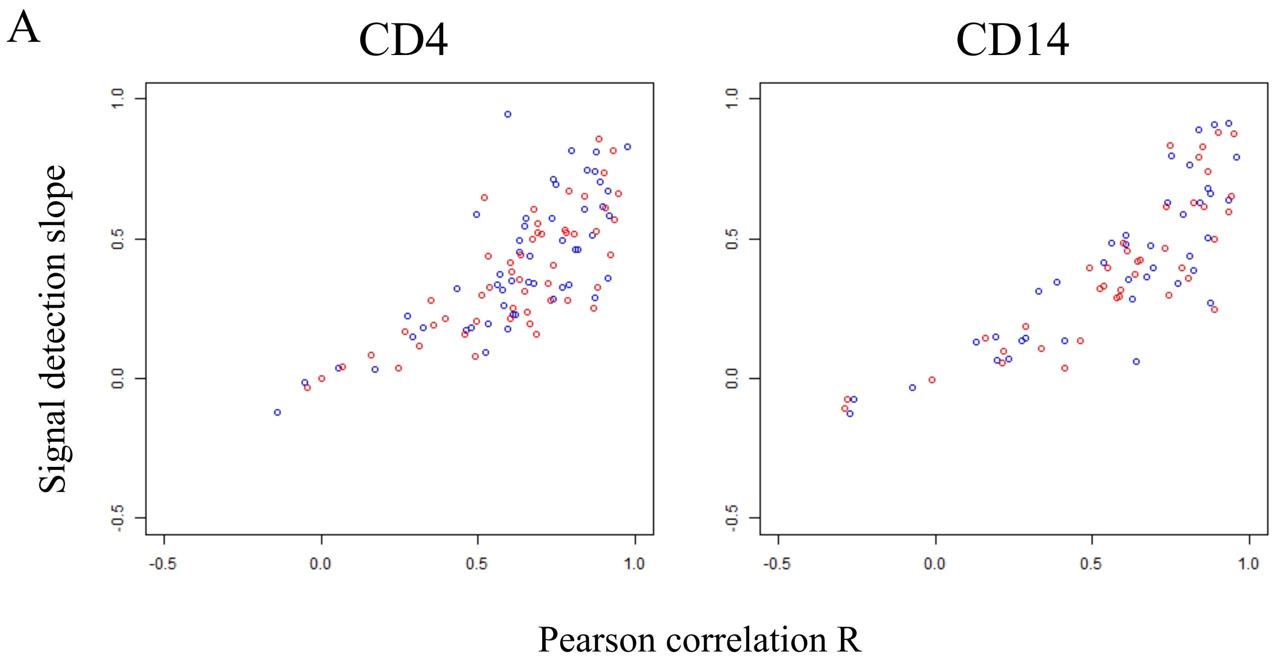

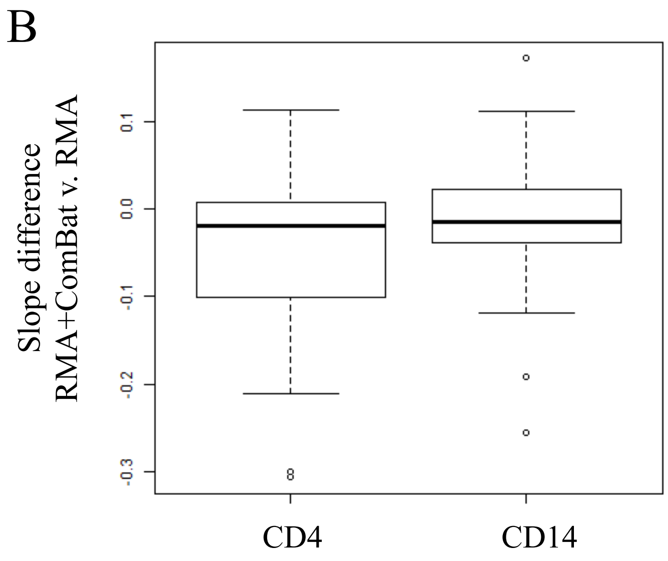

Effect of batch correction on signal detection accuracy. **A**) Signal detection slope is plotted versus inter-platform correlation as in Figure 2A: blue = RMA- and red = RMA +ComBat-preprocessed microarray expression values. **B**) Signal detection slope of expressed genes across samples in nCounter versus RMA-preprocessed microarray datasets was subtracted from the same signal detection slope in nCounter versus RMA-preprocessed and ComBat-corrected microarray datasets. Boxplots depict these differences in CD4 and CD14 datasets to indicate the effect of batch correction on signal detection accuracy.
