# Supplementary material for: Comparison of gene expression microarray data with count-based RNA measurements informs microarray interpretation
Source: BMC Genomics. 2014 Aug 4;15(1):649. doi: 10.1186/1471-2164-15-649 (PMC4143561; doi:10.1186/1471-2164-15-649)
Supplement: Supplementary file 11 — Additional file 11:: Noise v expression value. Comparison of noise versus microarray expression value. A) For each unexpressed gene, the standard deviation of log-ratios of all pairs of samples from RMA + ComBat- (CD4 and CD14) or RMA-preprocessed (CD16) microarray data is plotted versus the gene’s median microarray expression value. B) As (A) for invariant genes. (PDF 81 KB) [file 12864_2014_6367_MOESM11_ESM.pdf]

Additional File 11

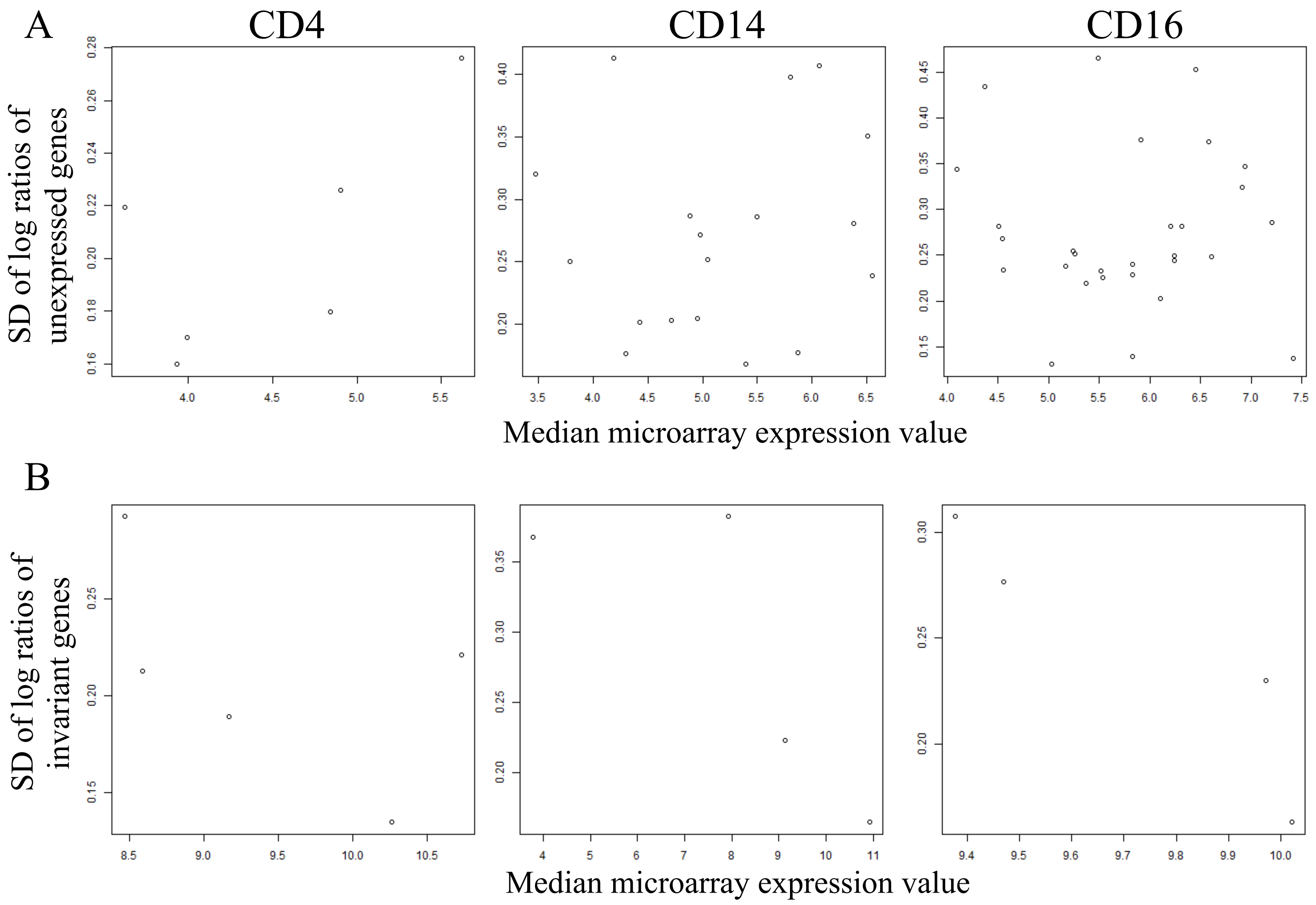

Comparison of noise versus microarray expression value. A) For each unexpressed gene, the standard deviation of log-ratios of all pairs of samples from RMA+ComBat- (CD4 and CD14) or RMA-preprocessed (CD16) microarray data is plotted versus the gene's median microarray expression value. **B**) As (A) for invariant genes.
