# Supplementary material for: Comparison of gene expression microarray data with count-based RNA measurements informs microarray interpretation
Source: BMC Genomics. 2014 Aug 4;15(1):649. doi: 10.1186/1471-2164-15-649 (PMC4143561; doi:10.1186/1471-2164-15-649)
Supplement: Supplementary file 12 — Additional file 12:: Mean expression histograms. Mean expression profiles: Histograms depict mean RMA + ComBat- (CD4 and CD14) or RMA-preprocessed (CD16) microarray expression values from full microarray datasets. (PDF 65 KB) [file 12864_2014_6367_MOESM12_ESM.pdf]

Additional file 12

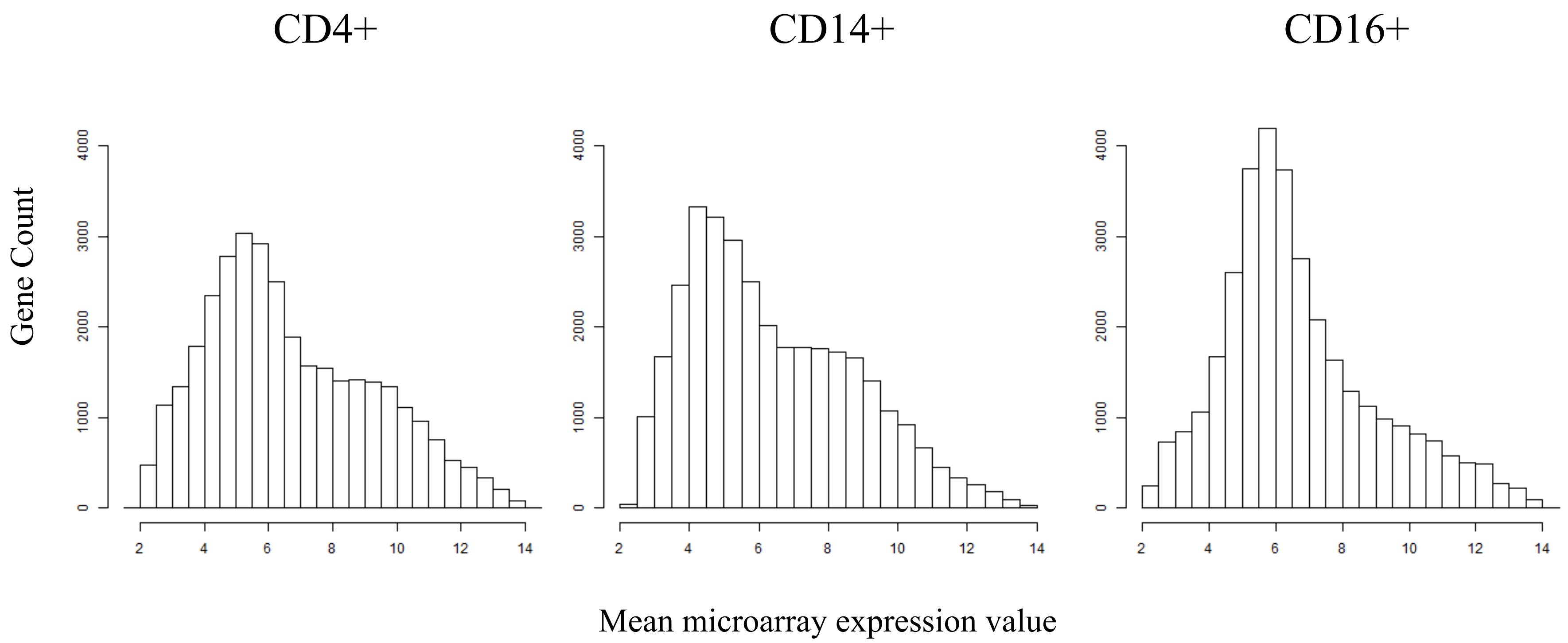

Mean expression profiles. Histograms depict mean RMA+ComBat- (CD4 and CD14) or RMA-preprocessed (CD16) microarray expression values from full microarray datasets.
